# Routes to control diffusive pathways and thermal expansion in Ti-alloys

**DOI:** 10.1038/s41598-020-60038-x

**Published:** 2020-02-20

**Authors:** Matthias Bönisch, Mihai Stoica, Mariana Calin

**Affiliations:** 1 Institute for Complex Materials, IFW Dresden, D-01069 Dresden, Germany; 2 Laboratory of Metal Physics and Technology, Department of Materials, ETH Zürich, CH-8093 Zürich Switzerland; 3 Present Address: Department of Materials Engineering, KU Leuven, B-3001 Leuven Belgium

**Keywords:** Mechanical engineering, Mechanical properties, Metals and alloys

## Abstract

β-stabilized Ti-alloys present several unexplored and intriguing surprises in relation to orthorhombic α″ phases. Among them are (i) the diffusion-controlled formation of transitional α″_iso_, α″_lean_ and α″_rich_ phases and ii) the highly anisotropic thermal expansion of martensitic α″. Using the prototypical Ti-Nb system, we demonstrate that the thermodynamic energy landscape reveals formation pathways for the diffusional forms of α″ and may lead to a stable β-phase miscibility gap. In this way, we derive temperature-composition criteria for the occurrence of α″_iso_ and resolve reaction sequences during thermal cycling. Moreover, we show that the thermal expansion anisotropy of martensitic α″ gives rise to directions of zero thermal strain depending on Nb content. Utilizing this knowledge, we propose processing routes to achieve null linear expansion in α″ containing Ti-alloys. These concepts are expected to be transferable to other Ti-alloys and offer new avenues for their tailoring and technological exploitation.

## Introduction

**Figure 1:**
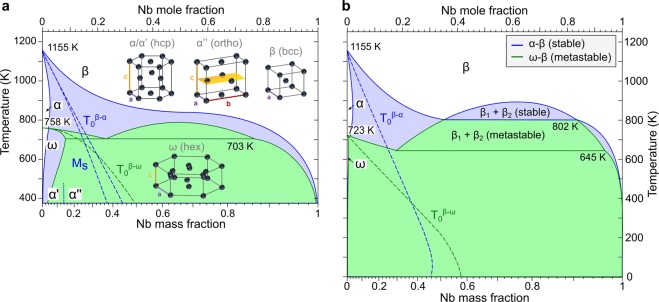
Calculated Ti-Nb phase diagrams. (**a**) Adapted from Zhang, Liu and Jin^22^, (**b**) calculated using the *TiGen* database^24^. The stable α-β diagram is shown in blue and on top of it the metastable ω-β diagram is drawn in green. Solid lines indicate phase boundaries if diffusion is allowed and dashed lines those without diffusion. The crystal structures are illustrated as insets in (**a**).

β-stabilized Ti-alloys provide the basis for a multitude of future applications in biomedicine and aeronautics^1,2^. Their low elastic moduli, shape memory and superelastic effects, blended with pronounced hardenability and attractive ductility captivates the attention of engineers and material scientists alike. Despite their seemingly simple phase diagrams β-stabilized systems, such as Ti-Nb (Fig. 1) show a vast array of phase transformations and transient states, attributed to the extensive α-β two-phase field. The ensuing exceptionally pronounced metastability necessitates large diffusional compositional changes and extended aging durations (up to many weeks or months) to reach (meta)stable equilibrium.

The nature of compositional fluctuations decisively influences the refinement of precipitation products^3–5^ and the β ↔ α″ martensitic transformation^6^. For instance, detailed (micro)structural studies uncovered non-conventional transformation pathways for α-precipitation^3,7–9^. Of particular note is the discovery of transitional α″-like structures (α″_iso_, α″_lean_, α″_rich_) during martensite decomposition and prior to α precipitation^7–11^. Furthermore, recently extraordinarily large anisotropic thermal expansion was revealed in α″ martensite of the Ti-Nb system^9,12^.

The successful development of novel structural and functional Ti-alloys with bespoke mechanical and shape memory behaviour depends critically on accurate descriptions of precipitation and decomposition processes. In addition, key for dimension critical components is the ability to control thermal expansion^13,14^. Nowadays, the core challenge in developing new Ti-alloys lies in unravelling the complexity of phase reactions and in deriving robust thermodynamic and structural descriptions to assist the alloy design process. Empirical trial-and-error methods still prevail today, nevertheless systematic attempts are made to develop and employ predictive capabilities through ab-initio^15–19^, phase-field^20,21^ and CALPHAD^22–27^ methods.

The present treatment takes a novel perspective to predict temperature-induced structural changes and phase transformations in Ti-Nb alloys from the Gibbs free energy landscape with primary focus on martensitic alloy formulations. We calculate the binary Ti-Nb phase diagram from the recently published *TiGen* database^24^. It turns out, the *TiGen* formulation differs substantially from an earlier formulation^22^ by giving rise to a stable miscibility gap in the β-phase. Informed by the Gibbs free energetics we explore precipitation pathways involving diffusion-mediated α″ phases. In this way, we delineate and compare against each other α″_iso_ formation and α″ martensite decomposition. By correlating the predicted pathways with experimental observations from synchrotron X-ray diffraction (SXRD) and Differential Scanning Calorimetry (DSC) these results can be conclusively rationalized, evidencing the efficacy of the presented strategy. In addition, we then examine the thermal expansion anisotropy of α″ martensite and propose 3 approaches to control thermal expansion in macroscopic polycrystalline Ti-alloy components by exploiting the thermal anisotropy on the single crystal level. These outcomes may guide the design of next-generation Ti-alloys and parts manufactured thereof.

## Results

### The quest for a faithful thermodynamic description for Ti-Nb

Based on the recent assessment by Yan and Olson^24^ we calculated the pertaining phase diagram (see Methods for details) and compare the results to the assessment by Zhang, Liu and Jin^22^ in Fig. 1. Crucially, in contrast to the assessment by Zhang, Liu and Jin^22^ a stable miscibility gap is predicted in the β-phase, similar to Ti-V, Ti-Mo and Ti-W. A stable miscibility gap is contingent on adequately high Gibbs free energies of competing phases under consideration (α and ω in the present case) in addition to the signs of the Redlich-Kister coefficients ^*β*^*L* > 0, Eq. (1). On the other hand, the emergence of a metastable miscibility gap, as present in Fig. 1a, depends solely on the signs of the Redlich-Kister coefficients, i.e. if one ^*β*^*L* is positive phase separation occurs. Therefore, two factors are responsible for the stable miscibility gap in Fig. 1b compared to Fig. 1a. (i) the depressed α-β transus at low Nb content ascribed mainly to the relatively larger ^*α*^*L*_0_ and (ii) the larger (positive) β-interaction parameters (Table 1). Because ^*β*^*L*_1_ > 0 the miscibility gap is asymmetric and shifted towards the Nb-rich side. Further, the ω-α equilibrium in Ti in Fig. 1b is 35  K lower than in Fig. 1a, resulting in a reduced temperature for the eutectoid point β_1_ → ω + β_2_. Both diffusionless equilibrium temperatures *T*_0_^*β-α*^ and *T*_0_^*β-ω*^ are lower than in Fig. 1a, especially *T*_0_^*β-ω*^ is strongly suppressed in the *TiGen* database relative to^22^. The existence of a stable miscibility gap in Ti-Nb, as predicted in Fig. 1b and hypothesized early on^28^, lacks definite experimental verification and remains speculative. We note that the underlying *TiGen* database^24^ was optimized using low-temperature data and, as the database authors remark, may not be accurate at high temperature. Therefore, we base the following treatment on the thermodynamical Ti-Nb assessment by Zhang, Liu and Jin (^22^, Fig. 1a). This description has proven itself suitable across a wide temperature range in previous studies^29,30^.

**Table 1:** Thermodynamic interaction parameters for the Ti-Nb system.

	β	α	ω	Ref.
^*ϕ*^*L*_*0*_	13045.3	11742.4	−3775.9	^22^
^*ϕ*^*L*_*0*_	14000	17200–4 *T*	16369 + 5.78 *T*	^24^
^*ϕ*^*L*_*1*_	2500	—	—	^24^

### Gibbs energetics: ω-α cascade vs. α″_iso_ formation vs. α″ decomposition

**Figure 2:**
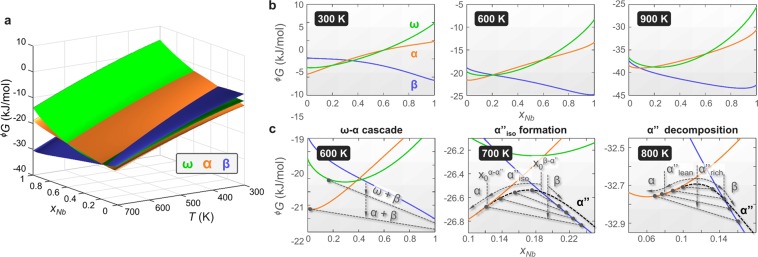
Free enthalpy landscape of Ti-Nb α, β and ω-phases calculated using the thermodynamic parameters in Zhang, Liu, Jin^22^. (**a**) Overview of effect of temperature on the free energy relations. (**b**) Sections of the free enthalpy landscape at constant temperatures over the full composition space. (**c**) Illustrations of ω-assisted α-precipitation, α″_iso_ formation and α″ decomposition. Grey dashed straight lines indicate (meta)stable tie lines. Arrows show the system’s path from the metastable initial state towards (meta)stable equilibrium.

Figure 2 shows the free enthalpy landscape of α, β and ω-phases for Ti-Nb depending on temperature and composition based on the description by Zhang, Liu and Jin^22^. Phase transformations, may they be martensitic or diffusion-based, and associated precipitation processes are rooted upon the relative stability of the phases involved; the free enthalpy surfaces in Fig. 2a delineate the corresponding energetics for Ti-Nb.

Figure 2b illustrates snapshots of the free enthalpy curves for selected temperatures. Informed by these, it is straight-forward to identify energetically favourable transformation sequences (i.e. likely reactions): Any likely reaction is synonymous with a spontaneous reaction and as such reduces the free enthalpy. On quenching a given alloy from the β-phase field, the phase exhibiting the lowest free enthalpy at the quench end temperature forms. When re-heated, the quenched metastable state is subject to contending compositional instabilities depending on the β-stabilizer content, temperature and exposure time. These instabilities are driven by gradual reductions in the free enthalpy facilitated by compositional changes of the constituents (α-, ω-, β-phases, martensites α′ and α″). Depending on composition and temperature the Gibbs free energy landscape forecasts several different precipitation and decomposition processes. Below we concentrate our discussion on 3 of them: (i) the precipitation of ω_iso_ and α, labelled here ω-α cascade, (ii) α″_iso_-formation and (iii) α″ decomposition; Fig. 2c illustrates the underlying Gibbs free energetics.

*ω-α cascade*: Metastable β, produced by quenching or martensite reversion, may precipitate ω_iso_ and α phases to reach equilibrium (Fig. 2c left). This precipitation cascade (with and without intermediate formation of ω_iso_) can be triggered almost within the entire α-β two phase field. It is an already well-established route for material engineers to strengthen β-stabilized Ti-alloys^31–33^. Due to its importance and relatively easy triggerability it is being widely researched from experimental and theoretical (modelling) point of views^5,20,27,34,35^.

α″_*iso*_*-formation*: If intermediate ω formation is energetically unfavourable, orthorhombic α″_iso_ may form instead before α appears. While a handful of studies observed α″_iso_ in different β-stabilized Ti-alloys^7,11,36,37^, the present treatment provides for the first time an intuitive explanation for its occurrence based on the system’s free energy landscape. This situation is illustrated in Fig. 2c (centre). When ω is energetically unfavourable, α″_iso_ provides a low energy pathway for Nb-depleted domains to transition from the β-phase to α-phase. Quite clearly, occurrence of α″_iso_ is mediated by the diffusional flux of Nb in contrast to the diffusionless formation of α″ martensite during quenching. Recent *in-situ* measurements of the α″_iso_ lattice parameters have revealed that they gradually evolve into those of hexagonal α^9^. α″_iso_ is therefore a partially transformed intermediate structure found between the reaction end members, parent β and product α. The displacive component that defines the orthorhombic geometry of α″_iso_ is linked in this way to its specific chemistry, in a similar manner as was proposed for the {222}_β_ plane collapse leading to ω_iso_^38^. In this sense, it seems justified to classify the formation of α″_iso_ as a mixed-mode diffusive-displacive phase transformation akin to ω_iso_ formation from β^38^. It is important to point out that to date no independent thermodynamic description for α″ exists and the ^α″^*G* curves in Fig. 2c consequently represent educated guesses as detailed below. For purposes of calculating *T*_0_^*β-α″*^ the Gibbs free energy of α″ is, for the lack of a more suitable formulation, commonly approximated through ^*α″*^*G* ≅ ^*α*^*G* (^24,22^ and Fig. 1). While this approach is tenable for low solute content where α″ strongly resembles α structurally, it becomes contentious at high solute content where α″ more closely resembles β. Instead, at high solute content we propose to approximate ^*α''*^*G* by ^*β*^*G* + *A* where *A* is a positive value reflecting the difference between ^*α″G*^ and ^*β*^*G* in concentrated alloys. This ensures that ^*β*^*G* < ^α″^*G* in martensite forming alloys and agrees with the free energy representation put forward by Davis, Flower and West^39^. At this point we would like to draw attention to the behaviour of ^α″^*G* at the intersections with ^*α*^*G* and ^*β*^*G*. Early and recent studies have shown that the lattice parameters and specific volume vary smoothly across the α′-α″ transition at ${x}_{0}^{\alpha -{\alpha }^{{\prime\prime} }}$ and that hexagonal α′ and orthorhombic α″ do generally not coexist^23,39–42^. In contrast, the transition from β to α″ around ${x}_{0}^{\beta -{\alpha }^{{\prime\prime} }}$ involves a strong disruption in crystal symmetry that carries forward an expansion of the specific volume^23,42^. Thus, an accurate thermodynamic depiction of the experimentally observed (dis)continuity requires a smooth behaviour (equal slopes) of ^*α*^*G* and ^α″^*G* at ${x}_{0}^{\alpha -{\alpha }^{{\prime\prime} }}$ and a discontinuous behaviour (unequal slopes) of ^α″^*G* and ^*β*^*G* at ${x}_{0}^{\beta -{\alpha }^{{\prime\prime} }}$. Metastable β may then - once kinetics permit - nucleate α″_iso_ which then continuously evolves into α by rejection of Nb.

From the thermodynamic landscape we derive a temperature criterion in terms of a necessary condition for α″_iso_ occurrence. Assuming that α″_iso_ formation is conditional on ^α″^*G* < ^*ω*^*G* around ${x}_{0}^{\alpha -\beta }$, the minimum temperature for α″_iso_ formation is the temperature for which the Gibbs free energies ^*α*^*G*, ^*β*^*G* and ^*ω*^*G* at ${x}_{0}^{\alpha -\beta }$ are identical, i.e. they intersect at a single point. This temperature is 631  K (358 °C) as derived from^22^. Above 631  K, ω is energetically not favoured relative to α″ and the lowest energy path from β to α leads via α″_iso_. Further, the alloy content of β must be above ${x}_{0}^{\beta -{\alpha }^{{\prime\prime} }}$. As we will see later these conditions reflect well the experimental observations.

α″*-martensite decomposition*: Figure. 2c (right) illustrates the martensite decomposition process in solute lean Ti-Nb. In contrast to α″_iso_ formation which starts from metastable β, α″ has lowest free energy at the decomposition start. Due to the negative curvature of ${}^{\alpha {}^{{\prime\prime} }}G$ around the start composition (indicated by the vertical grey arrow, $\frac{{\partial }^{2}{}^{{\alpha }^{{\prime\prime} }}G}{\partial {{x}_{Nb}}^{2}} < 0$) small compositional fluctuations become amplified. In this way Nb depleted (α″_lean_) and Nb enriched (α″_rich_) domains form, which evolve continuously into equilibrium α and β phases. Interestingly, even though conceptualized for the first time more than 35 years ago^10,39,43^, α″ decomposition has only recently come into focus^8,9,44,45^. Its investigation has benefited significantly from the use of high temperature *in-situ* diffraction set-ups at synchrotron facilities^8,9^, similarly to the study of α″_iso_. Most notably, while *ex-situ* XRD in earlier work struggled to detect variations of the α″_lean_ lattice parameters with holding time^25^, *in-situ* measurements clearly revealed their evolution towards α^9^.

At this point, we would like to draw the reader’s attention to the metastability pertaining to all athermal and diffusion-mediated forms of α″. This contrasts with the Gibbs energetics in ternary Ti-Al-Nb and related Ti-Al-X systems (X denoting a β-stabilizer), where chemically ordered orthorhombic O-phase is thermodynamically stable and appears as an equilibrium phase^46,47^.

### Reaction sequences during thermal cycling reinterpreted

**Figure 3:**
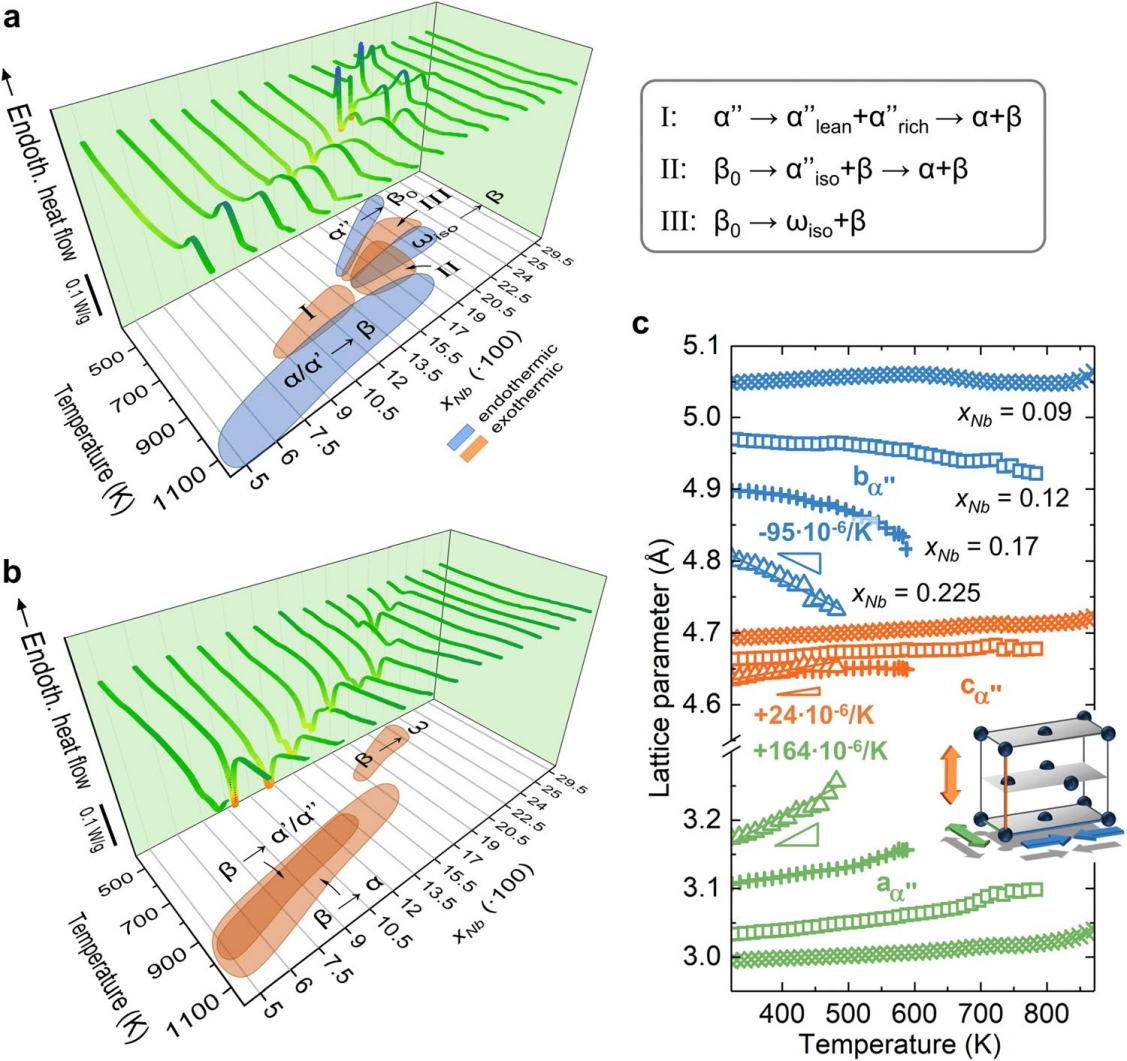
Temperature-induced structural changes in Ti-Nb alloys. Role of Nb content on transformation sequences triggered by (**a**) heating the quenched state and by (**b**) cooling from the β-phase field. Data for *x*_*Nb*_ = 0.075–0.295 in (**a**) and (**b**) were recompiled from^29,30^. For visualization purposes values of *x*_*Nb*_ in (**a,b**) are multiplied by 100. (**c**) Variation of lattice parameters of α″ martensite with temperature. Of the 4 compositions illustrated, *x*_*Nb*_ = 0.225 exhibits the largest positive and negative expansion rates. Data in (**c**) are replotted from^9^.

Ti-Nb alloys undergo a series of transformation events when temperature-cycled in the martensitic state, as illustrated in Figs. 3a,b. Alloy composition plays a central role in determining the transformations’ nature and sequence^29,30^. Here we restrict our discussion to salient features of α″-martensitic alloys of intermediate Nb content (9–19  at.%) in the context of the foregoing analysis of the thermodynamic landscape. The Gibbs free energy relationships (Fig. 2) predict the formation of intermediate orthorhombic products α″_iso_, α″_lean_ and α″_rich_ for appropriate temperature and composition regimes. In recent experiments it was possible to detect and follow these transitional structures in binary Ti-Nb *in-situ* by high temperature XRD^9^. By now, transitional orthorhombic phases have been observed in several β-stabilized Ti-alloys including a few commercially important formulations^7–11^. Informed by the energy landscape and the experimental evidence^9^ we reinterpret the heat flow signatures of 9–19 at% Nb alloys recorded by DSC (Figs. 3a,b). The exothermic peak I (Fig. 3a) between 700–900  K in 9–13.5 at% Nb is thus attributed to martensite decomposition α″ → α″_lean_ + α″_rich_ → α + β mediated by Nb-enriched (α″_rich_) and Nb-impoverished (α″_lean_) α″. In the higher solute alloys of 15.5–19 at% Nb above α″ → β_0_ martensite reversion and below the α-β transus, α precipitation from β occurs via transitional α″_iso_, viz. β_0_ → α″_iso_ + β → α + β. This reaction was observed over a broad temperature range of up to ~200  K^9^ in parallel with the dissolution of ω_iso_ into β around 758  K. Its broad exothermic signal is therefore largely superimposed by the endothermic signal of ω_iso_ → β. Appreciating the uncertainty of the thermodynamic description of α″ we note that the temperature and composition regimes in which α″_iso_ occurs correspond very well with the conditions derived from the Gibbs energy landscape previously. Furthermore, the occurrence of β → α during cooling from the β-phase field depends on the kinetics. For instance, in the present case β → α occurred for *x*_*Nb*_ ≤ 0.17 while it was suppressed for higher Nb content (Fig. 3b).

Findings like these motivate the development of thermodynamic models with predictive capabilities for assessing and tailoring thermal protocols. It largely remains to be seen how α″ martensite decomposition and α″_iso_ formation can be effectively exploited to improve mechanical and functional properties of Ti-alloys. Promising effects were reported for Ti-V and Ti-Mo in the early days of the exploration of Ti-alloys, inasmuch they experience substantial strengthening when aged by spinodal decomposition of α″-martensite^25,43^.

### Thermal expansion control in Ti-alloys

**Figure 4:**
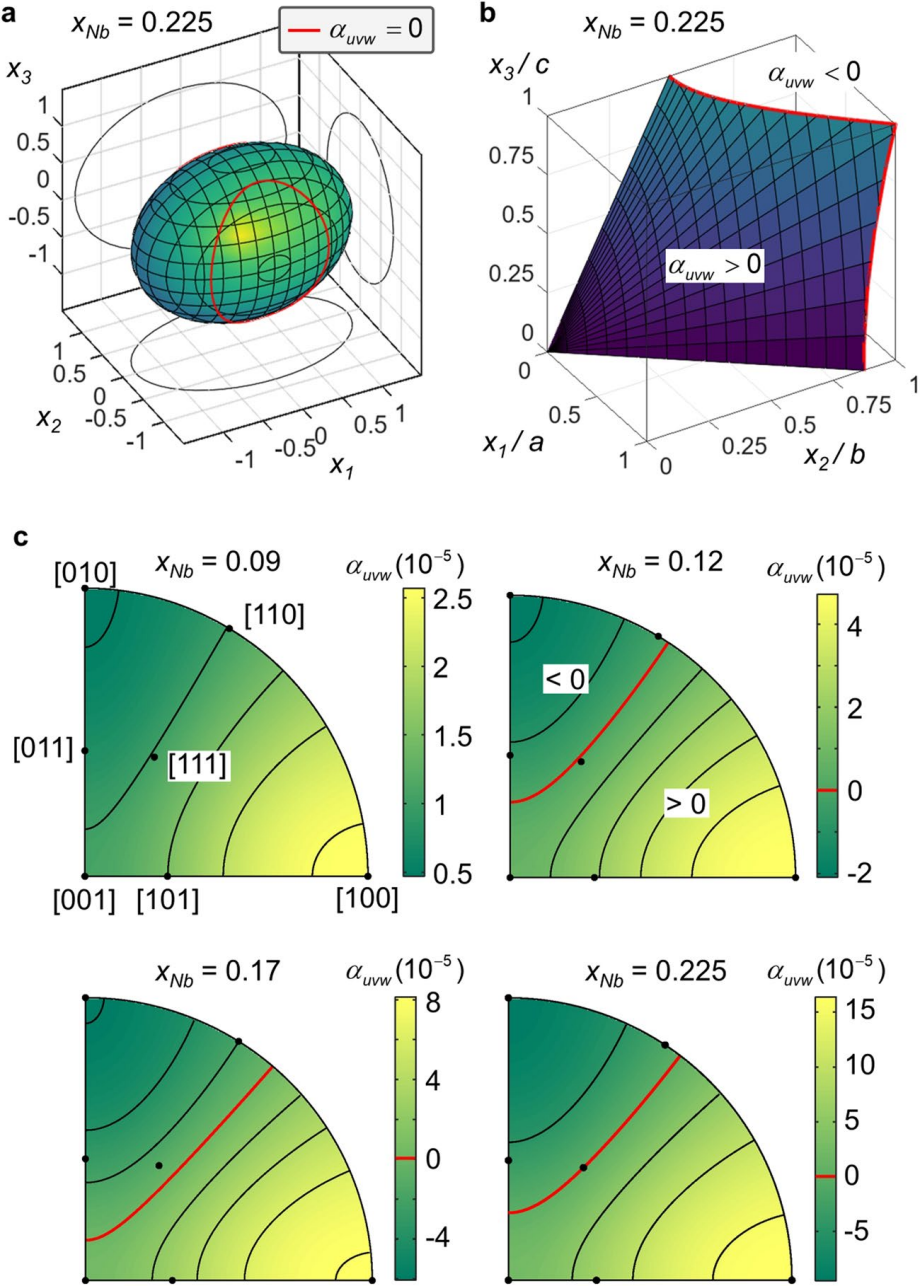
Anisotropic thermal expansion in Ti-Nb alloys. (**a**) Thermal expansion ellipsoid and (**b**) the cone of zero stretch directions inside the α″ unit cell for Ti_77.5_Nb_22.5_. Directions off the cone either contract or expand. For visualization purposes the thermal expansion coefficients in (**a**) were scaled by a factor of 2000. (**c**) Stereographic projections of the thermal expansion for orthorhombic martensite α″ depending on the Nb content. Directions of zero stretch are marked red.

Recently, giant and highly anisotropic linear thermal expansion rates were uncovered in solute rich α″ martensite of Ti-Nb^9,12^. These are illustrated in Fig. 3c by the response of the orthorhombic unit cell to a temperature change as derived from *in-situ* SXRD. When heated *a* lengthens drastically and *b* shortens similarly strongly; *c* lengthens only weakly. A unit sphere of a hypothetical martensite single crystal thus gets distorted into an ellipsoid, Eq. (2), as showcased in Fig. 4a for *x*_*Nb*_ = 0.225. Importantly, due to the contraction along *b* for *x*_*Nb*_ > 0.09 the thermal expansion of α″ becomes zero for particular crystallographic directions of the unit cell (i.e. *α*_*uvw*_ = 0 for certain [uvw], see Eq. (3)). The unstretched directions form an elliptical cone about *b* (Eqs. (4) and (5)), which is exemplarily illustrated in Fig. 4b for *x*_*Nb*_ = 0.225 inside a single α″ unit cell. It runs from approximately [012] over [111] to [110]. This behaviour is hardly affected by composition albeit the alloy content sensitively projects onto the expansion magnitude, as demonstrated in Fig. 4c. Larger expansion rates correlate with higher Nb content leading to exceptionally large stretches and contractions observed for *x*_*Nb*_ = 0.225 across more than 150 K. Only for *x*_*Nb*_ = 0.09 deviating behaviour is found, to the extent that directions of zero expansion are absent since all its expansion coefficients are positive. The volumetric expansion rates for the 4 alloys presented are positive and range between *α*_*V*_ = 24.7–91 ppm/K. Together with the contraction along *b* this provides a remarkable materials design situation inasmuch it opens the door to bespoke thermal expansion properties through texture and composition control. Taking advantage of the described thermal expansion anisotropy in α″ we propose 3 approaches to engineer zero thermal expansion in Ti-alloys:

i) Single crystal-like textures: By synthetizing α″ microstructures with a very strong texture approaching that of a single α″ variant, zero expansion is obtained along directions *α*_*uvw*_ = 0 (see Methods) and illustrated by Fig. 4. This approach has the benefit of yielding low thermal stresses across interfaces of adjacent grains.
ii) Introduction of a [010]_α″_ texture component: Starting from a random initial orientation distribution function, texture components favouring the alignment of [010]_α″_ (or a nearby contracting direction) along a specific sample direction are introduced. By adjusting this component’s strength via e.g. imposing the required level of deformation the initially isotropic linear expansion of *α*_*V*_*/3* can be reduced to zero along this sample direction.
iii) Controlling the α″ volume fraction: An appropriate volume fraction of α″ is introduced into a parent single- or multi-phase microstructure and α″ is intentionally oriented or limited to a single variant (e.g. by external stress). Likewise, α″ may be combined with other alloys or materials in a composite fashion to directionally compensate thermal expansion. Similar to ii), null thermal expansion may be obtained for sample directions co-linear with contracting directions in α″ (i.e. for which *α*_*uvw*_ < 0).

The controlled creation of crystallographic textures of α″ is central to all 3 approaches. Yet, to date deformation textures in Ti-alloys consisting to a large part or completely of α″ have been rarely studied. For rolling of α″-martensitic Ti-Nb, it was reported that [010]_α″_ aligns along the rolling direction^12,48^. Consequently, by taking approach ii and by adjusting the rolling thickness reduction, zero temperature-induced length change along the rolling direction may be obtained^12^. Furthermore, compositional fluctuations in the β-phase - which are often considered a nuisance - could instead help control the volume fraction and spatial distribution of mechanically induced α″ when following approach iii. In this way, tuning of the linear thermal expansion was recently demonstrated in Ti‒24 Nb‒4Zr‒8Sn^49^. In future, to be able to successfully exploit the thermal expansion anisotropy solid knowledge of formation and manipulation of α″ textures will be necessary. This calls for detailed experimental studies of texture formation in α″ containing alloys.

## Discussion

The development of an accurate thermodynamic description for β-stabilized Ti-alloys that is valid both at low and high temperatures remains a challenging task. Our calculations demonstrated that the question about the existence of a stable β miscibility gap in Ti-Nb is not answered consistently by the current models and should receive special attention in future modelling efforts. Overall, composition and temperature trends of pathways triggered experimentally (ω-α cascade, α″_iso_ formation, α″ decomposition) follow those anticipated by Gibbs free energy calculations. These findings encourage to consult the alloys’ energetics more regularly during experimental composition and processing design. Furthermore, the highly anisotropic thermal expansion of α″ martensite gives rise to zero thermal stretch along specific directions in the orthorhombic unit cell. Most relevantly, through customizing the crystallographic texture and α″ volume fraction null thermal expansion can be obtained in polycrystalline aggregates. Summarizing, this work offers new design avenues for novel single and multi-phase Ti-alloys and invites to further explore these versatile materials.

## Methods

### Material preparation and characterization

Experimental results presented are for binary Ti-Nb alloys fabricated through arc-melting and cold crucible casting followed by homogenization at 1273  K for 24  h and water quenching. Phases and their structural characteristics were studied with transmission X-ray diffraction (XRD). Thermal analysis was carried out via temperature cycling at constant heating and cooling rate using Differential Scanning Calorimetry (DSC). Temperature dependent lattice parameters were derived from *in-situ* synchrotron XRD. In detail descriptions of the alloy preparation and characterization are given in^9,29,41^.

### Thermodynamic calculations

Thermodynamic calculations served two purposes: (i) examine and validate experimentally observed transformation pathways against the system’s thermodynamics in terms of the participating phases’ free enthalpy curves; (ii) derive the Ti-Nb phase diagram from the recently published *TiGen* database^24^. Within the current scope two differing thermodynamic descriptions were employed; that by Zhang, Liu and Jin from 2001^22^ and that by Yan and Olson (*TiGen* database) from 2016^24^. Each phase (indicated by *ϕ*) is modelled as a disordered substitutional solid solution with its molar Gibbs free energy formulated as

1 $${}^{\phi }G={x}_{Nb}{}^{\phi }G_{Nb}+{x}_{Ti}{}^{\phi }G_{Ti}+RT({x}_{Nb}\,\mathrm{ln}\,{x}_{Nb}+{x}_{Ti}\,\mathrm{ln}\,{x}_{Ti})+{}^{\phi }L_{0}{x}_{Nb}{x}_{Ti}+{}^{\phi }L_{1}{x}_{Nb}{x}_{Ti}({x}_{Nb}-{x}_{Ti})$$

^*ϕ*^*G*_*Nb*_ and ^*ϕ*^*G*_*Ti*_ are the Gibbs free energies of the unary systems (Nb and Ti), *R* the gas constant, *T* the absolute temperature; *x*_*Nb*_ and *x*_*Nb*_ = 1 − *x*_*Nb*_ denote the mole fractions of Nb and Ti. Unary Gibbs free energies of α and β above 298.15  K are based on the *SGTE* database^50^ and those below 298.15  K follow the expressions by Vřešt’ál, Štrof and Pavlů^51^. ^*ω*^*G*_*Nb*_ and ^*ω*^*G*_*Ti*_ are constructed relative to ^*β*^*G*_*Nb*_ and ^*α*^*G*_*Ti*_, respectively:

$${}^{\omega }G_{(Nb,Ti)}={}^{(\beta ,\alpha )}G_{(Nb,Ti)}+{u}_{0}+{u}_{1}T+{u}_{1}/T$$

The corresponding coefficients *u*_*i*_ are provided in Table 2. For ^*ω*^*G*_*Ti*_ below 298.15  K Yan and Olson used an expression different from Eq. (1) formulated independently of ^*ω*^*G*_*Ti*_^24^. Chemical interactions between Ti and Nb are accounted for by the excess Gibbs free energy expressed as a Redlich-Kister polynomial (last 2 terms in Eq. (1)) with interaction parameters ^*ϕ*^*L*_0_ and ^*ϕ*^*L*_1_^52^; Table 2 lists them. While Zhang, Liu and Jin used strictly regular descriptions (^*ϕ*^*L*_*0*_ *T*-independent) for all phases, Yan and Olson proposed a subregular description with constant interaction parameters for β and *T*-dependent ^*ϕ*^*L*_*0*_ for α and ω.

**Table 2:** Polynomial coefficients for ^*ω*^*G*_*Nb*_ and ^*ω*^*G*_*Ti*_.

	u_0_	u_1_	u_2_	Ref.
^ω^G_Ti_	1886.7	−0.1561	—	^22^
^ω^G_Nb_	15000	2.4	—	^22^
^ω^G_Ti_	−1401.86	4.439080	110185.9	^24^
^ω^G_Nb_	6878	—	—	^24^

To analyse precipitation and decomposition pathways against the system’s energetics, free enthalpy plots for α, β and ω were calculated at selected temperatures. To calculate the Ti-Nb phase diagram from the *TiGen* database^24^ a customized code was developed in GNU Octave (www.octave.org,^53^) which determines equilibrium concentrations by finding common tangents to the ^*ϕ*^*G* curves. All thermodynamic parameters are given in J, mol and K.

### Thermal expansion anisotropy

Thermal expansion distorts a unit sphere of material into the ellipsoid

2 $$\frac{{{x}_{1}}^{2}}{{(1+{\alpha }_{1})}^{2}}+\frac{{{x}_{2}}^{2}}{{(1+{\alpha }_{2})}^{2}}+\frac{{{x}_{3}}^{2}}{{(1+{\alpha }_{3})}^{2}}=1$$

*x*_*1*_, *x*_*2*_ and *x*_*3*_ are the α″ crystal frame coordinates parallel to *a*, *b*, *c*, respectively, and the *α*_*i*_’s denote the pertaining expansion coefficients given by the thermal expansion tensor

$$[{\alpha }_{ij}]=[\begin{array}{ccc}{\alpha }_{1} & 0 & 0\\ 0 & {\alpha }_{2} & 0\\ 0 & 0 & {\alpha }_{3}\end{array}]$$

The magnitude of *α*_*ij*_ along the crystallographic direction [uvw] is^54^

3 $${\alpha }_{uvw}=\frac{{\alpha }_{ij}{x}_{i}{x}_{j}}{{|x|}^{2}}=\frac{{\alpha }_{1}{a}^{2}{u}^{2}+{\alpha }_{2}{b}^{2}{v}^{2}+{\alpha }_{3}{c}^{2}{w}^{2}}{{a}^{2}{u}^{2}+{b}^{2}{v}^{2}+{c}^{2}{w}^{2}}$$

with

$${x}_{1}=u\cdot a\,{x}_{2}=v\cdot b\,{x}_{3}=w\cdot c.$$

In the present case, *α*_*1*_ > 0, *α*_*2*_ < 0, *α*_*3*_ > 0 for *x*_*Nb*_ > 0.09. The unstretched directions then form an elliptical cone about *x*_*2*_ and are obtained as

4 $$\begin{array}{c}u=\frac{{n}_{1}}{a}\cdot \,\cos (t)\\ v=\pm \,\frac{1}{b}\sqrt{1-{{n}_{1}}^{2}\cdot {\cos }^{2}(t)-{{n}_{3}}^{2}\cdot {\sin }^{2}(t)}\\ u=\frac{{n}_{3}}{c}\cdot \,\sin (t)\end{array}$$

where $$t\in [0,2\pi ]$$ and

5 $${n}_{1}=+\,\sqrt{\frac{1}{1-{\alpha }_{1}/{\alpha }_{2}}}\,{n}_{3}=+\,\sqrt{\frac{1}{1-{\alpha }_{3}/{\alpha }_{2}}}$$

## Data Availability

The data on which the results of this study are based can be made available by contacting the corresponding author.
